# On Syphilitic Pains and Diseases of the Bones

**Published:** 1827-04

**Authors:** Cæsar Hawkins

**Affiliations:** Surgeon, and Lecturer on Anatomy in the School of Great Windmill-street.


### SYPHILIS.

### *On Syphilitic Pains and Diseases of the Bones.* By Cæsar Hawkins, Esq, Surgeon, and Lecturer on Anatomy in the School of Great Windmill-street.

*Recent investigations have elicited an immense number of facts and observations, from which it is sufficiently clear that there is no symptom of lues which may not be cured without mercury; but it remains so to arrange these facts, as to

* In a former part of this Journal, (Nos. 290 and 291,) I have described another form of syphilitic disease—viz. Ulcerations of the Larynx; on which sufficient attention did not appear to me to have been bestowed.

determine, on safe principles, under what circumstances, and in what forms of the disorder, we should have recourse to mercury; and when we ought to give the preference to other modes of treatment.

There can be no doubt that there are many symptoms which are usually considered as the direct consequences of inoculation with the syphilitic virus, but which, in fact, are only secondarily produced by it.

Some confusion of this kind has probably arisen upon the subject of those affections which are usually called *syphilitic pains*; for it is not otherwise easy to account for the fact, that, by the majority of the profession, mercury is still considered essentially necessary for every form and every stage of these disorders: or, if other medicines are used as substitutes for mercury, or to assist its operation, they are employed indiscriminately, and without reference to the nature and situation of the pains.

I am induced, however, to believe that a set of symptoms are commonly classed together under the comprehensive term *venereal pains,* which are essentially distinct from each other in their nature, and which are situated in perfectly different structures; that some of these are comparatively of little importance, while others are exceedingly dangerous in their course. If it be true, then, that this difference of kind exists, it becomes probable that a different mode of treatment will be required: hence an attempt to separate them, and to determine what remedies are appropriate to each form of the disorder, may not be without its use.

Syphilitic pains, or, more properly speaking, painful affections following syphilis, may be divided into four distinct species:

1st. The first are those which have their seat in muscles, and in their tendons and fasciæ.

2dly. Those which arise from disease of the synovial membranes and bursæ mucosæ.

3dly. Those which are situated in the fibrous structure of the ligaments, periosteum, and pericranium.

4thly. The pains which arise from affections of the structure of the bones themselves.

It is true that two, or even all, of these different affections often exist in the same individual at the same time, but it is equally certain that they arise separately in other cases; and discrimination appears to me to be of importance, because some of these symptoms are not strictly syphilitic, and do not require the use of mercury in any case, and the remedies best adapted to one form of pains are useless or injurious in others.

1. *The painful affection of the muscles, tendons, and fasciæ.*—This is a very common occurrence after a syphilitic sore, and is often the only symptom present: it is generally a very early symptom, and thus evidently differs from those other affections which Mr. Hunter describes as constituting the second stage of lues; and it is well designated by the patient—pains in all the limbs.

This must not be confounded with that pain and weariness of the muscles, which often precedes the appearance of a syphilitic eruption, and is commonly removed by the establishment of the eruptive disorder. This latter muscular pain resembles that which is felt in common catarrhal disorders, and is attended with symptoms of febrile excitement, in a much greater degree than in the painful affection to which I am alluding.

2. *Inflammation of synovial membranes.*—The next description of pains which require notice are those which arise from an affection of the synovial membranes of the different joints and bursæ, and the serous linings of the sheaths of tendons; this disease being, in general, readily distinguishable by the situation and swelling of the affected parts.

I shall not occupy the time of my readers by describing the symptoms of either of these two disorders, since they are minutely described in various works on Rheumatism. There is, in truth, no essential difference between the affections of the muscles and joints which occur after syphilis, and chronic rheumatism of the same structures arising from other constitutional causes. Their character and situation, and their immediate causes, seem to be the same in each: their obstinacy and tendency to return, and the little control of medicine over them in many cases, are circumstances which apply to the idiopathic as well as to the specific disease. The proper explanation, no doubt, is, that the irritation of the syphilitic virus upon the system,—the employment of mercury,—and the mental depression for the most part produced in those who labour under venereal disorders, act as predisposing causes, giving a tendency to the production of rheumatic pains, on the application of a proper excitant, which in almost all cases is exposure to cold and damp.

Exactly the same disease occurs after a venereal sore, when no mercury is exhibited,—when mercury is employed for the cure of primary symptoms,—when this medicine is given for other disorders,—and when neither the venereal poison nor its antidote have had any share in the production of the disease. There cannot, therefore, be any reason for retaining the specific name "venereal pains;" and, if so, those who are most attached to the mercurial plan of treatment in syphilis may agree with me, that, for these painful affections of the muscles and synovial membranes, mercury (at least in the form of a regular course,) is in no case to be considered necessary.

For the same reason that I abstained from a description of the symptoms of these diseases, it will be unnecessary to enter into a consideration of their treatment. I shall only remark, that it is in the painful affection of the muscles that guaiacum, which has been extolled for its general antisyphilitic virtues, is chiefly useful; and that, as a general rule, it will be found that sudorifics are most efficacious in these pains, while antiphlogistic remedies, particularly colchicum, together with alteratives, are of most service in the chronic affections of the joints and bursæ.

3. *Of the affection of the periosteum and ligaments.*—The affection of the periosteum varies so much, that no description can apply to every case. It is generally a late symptom of lues, but I have known it to occur as early as two months from the first reception of the poison. Sometimes the pain is very intense, at others scarcely any is felt. In some persons the formation of nodes occurs very early; in others, the pain lasts a considerable time, and no swellings are ever formed. At one time the pain is entirely local, or confined to one or two bones, or even parts of them; at another, every bone in the body shows the existence of the disorder. This is the case in a skeleton in the collection belonging to Mr. Mayo and myself in Great Windmill-street, in which even the vertebræ, pelvis, and phalanges of the fingers, bear some marks of the disease in the periosteum.

There is some difference in the progress of the disorder when it affects the pericranium, compared with the corresponding affection of the periosteum of other bones. This arises partly from the finer texture of the membranous covering of the cranial bones, but principally from the influence of the disease upon the dura mater and the brain. I propose, therefore, first to consider the symptoms and appearance of the disease in the periosteum of the bones of the body generally, and afterwards to describe its progress in the pericranium.

I. There is commonly a dull heavy pain in all the bones of the body, distinguished even by the patient himself from the muscular and synovial affection, by its deeper situation and more fixed character: this is frequently periodical, and the time at which the attack occurs is generally very early in the morning. There is considerable tenderness, which may be detected, even in bones the most deeply situated, by pressure steadily applied in the interstices of the muscles. Restlessness, emaciation, a quick and feeble pulse, indicate great irritability of the constitution, rather than any great febrile excitement.

If the bones are examined after these pains have existed some time, the periosteum is found to be slightly thickened, and more vascular than usual, and the contiguous surface of the bone has not its usual white and shining appearance. The shape of the bone is natural, and the surface is not raised, but when thus affected is slightly softened and more spongy, and consequently more rough, than in its natural state. There is no fluid effused, and the periosteum adheres to the bone with its usual firmness; so that it cannot be said that any ulcerative process has actually commenced, but there seems to be an irregularity in the action of the vessels, preparatory to the formation of caries. If the bones are dried, the same difference of colour and hardness is preserved, so as to give a very peculiar mottled appearance to the bones; these darker patches of a brown colour, and of irregular size and shape, being intermixed with other parts of the bone still possessing the natural white smooth surface. This is most striking in the shafts of the cylindrical bones, but is also apparent in the more spongy bones, as on the bodies of the vertebræ.

In this stage of the disorder, it might admit of discussion whether the disease originates in the periosteum, and the bone becomes secondarily affected, or whether the primary seat of the disease is in the external surface of the bone. Mr. Hunter is of the latter opinion; but, if we consider the usual course of diseases of the bones, and trace the farther progress of this particular disease in the formation of nodes, exostosis, and caries,—and if we observe the pain and tenderness of the entire periosteum, while this appearance of the bones is only partial, there can be little doubt, I imagine, that the periosteum is the structure which is first affected.

At the same time that this disordered action exists in the periosteum, it is not unusual to find the ligaments similarly affected, as we might naturally expect from the resemblance of their structure to that of the periosteum. There can be no difficulty, however, in distinguishing the pain and tenderness around the joints, and the thickening and induration of the ligaments, from the disease of the synovial membranes.

After the disease has existed thus universally for some time, particular portions of the periosteum become more indurated than the rest, and local tumors or nodes are produced. It is not unusual to divide nodes into two kinds, which are called soft and hard nodes; there being in the latter, a simple indurated swelling over the bones, in the former, fluid having been secreted under the periosteum. This distinction is not without its use, if our practice is influenced by it; for I believe it will invariably be found that, where matter is thus formed in the nodes, it indicates a low and cachectic state of the constitution, and consequently points out the danger of employing mercury. But such a division does not at all describe the nature of the tumor, nor its origin; for each of these terms is applicable to a swelling which begins in the periosteum, as well as to another arising from primary disease of the bone.

It is well known that the bones most subject to nodes are those which are most exposed, and that they are usually formed on those surfaces and in those parts which are only covered by the integuments; a circumstance which Mr. Hunter attributes to the joint effect of the application of cold and the hardness of the bone, believing that the bone is the original seat of the disease; but there is an equally perceptible difference in the periosteum of the shafts of the cylindrical bones, compared with that covering the more spongy extremities, to which the inferior liability of the latter parts to form nodes may, with as much propriety, be ascribed.

In a healthy constitution, the periosteum becomes gradually thickened in some particular part, so as to form a tumor more or less prominent; the periosteum remaining firmly united to the bone, but not contracting any adhesion to the integuments. Sometimes the tumors only rise at first during the nocturnal exacerbations, and disappear in the morning: in this stage there is probably only a vascular tumefaction, or some temporary secretion of fluid in consequence of the inflammation. The tumor rises gradually from the bone, and is perfectly uniform and smooth on the surface; being, by these circumstances, easily distinguishable from common exostoses.

If now cut down upon, the node is found to be composed distinctly of the thickened periosteum, having a semi-cartilaginous appearance, in which a few bony spicula are often imbedded. The bone beneath is at first perfectly healthy, but after some time a slight roughness of the surface may be perceived, from incipient ulceration of a healthy character; or the bone itself may rise into the tumor, the osseous growth being merely an increased thickness of the outer harder part of the bone. Irregular tumefactions of this kind are often felt, particularly on the tibia, which continue after all specific action has ceased in the system, and become wholly free from pain and inconvenience.

In a bad constitution, on the other hand, instead of hard tumors, composed of periosteum at first and afterwards of bone, a secretion of matter takes place very early in some of the nodes, between the periosteum and the bone, and an abscess is produced, containing a glairy kind of fluid, of a dark colour. The extent of inflammation in this species of node is often very trifling, so that the matter remains a long time under the periosteum without giving any pain, and without changing in quantity, and is often entirely absorbed. In other cases the skin becomes red, and unites with the periosteum; it then changes to a purple colour, and finally ulcerates, a ring of thickened periosteum being felt around the opening. Sometimes a very small opening takes place, and, as soon as the matter has been evacuated, a healthy action commences, and union is again produced between the bone and the periosteum. A small puncture made early with the lancet is often attended with the same beneficial result. In other cases, caries and exfoliation take place, the progress of which is nearly the same as in similar affections arising from other causes.

II. When the pericranium is affected, there is at first considerable pain in the head, which is followed in a short time by tenderness of the scalp, with slight puffiness, sometimes amounting to actual œdema. The pain is generally greatest in the forehead, where distinct nodes are often produced, which sometimes suppurate. In these respects the disease is similar to the affection of the periosteum in other parts of the body, as well as in the periodical attacks of the pain and the constitutional disturbance. But after some time other symptoms are added. The mouth is frequently observed to be drawn to one side; the sense of touch is diminished in the skin of the face; deafness is produced; the sense of taste is impaired; and the motions of the tongue, eyelids, and lips, are imperfectly performed. Then the faculties of the mind are found to be weakened; the memory is imperfect, and a languor and half-torpid state of the intellect is produced,—sometimes with constant drowsiness: but in some few cases a high state of excitement is present, which causes actual mania. There are occasionally fits resembling epilepsy, spasmodic cough, with convulsions of the limbs, increasing in frequency, but leaving the patient comparatively well in the intervals, till at last some severer fit carries him off suddenly, or he sinks as if from exhaustion.

The progress of the symptoms, which have been thus rapidly enumerated, may be divided into three distinct periods, with which the alteration of structure will be found to correspond. In the first stage, more or less thickening of the external covering of the bone may be perceived, which begins in the pericranium, but also affects the tendon of the occipito frontalis muscle, where they are in contact. The outer surface of the bone may be rough and irregular, and increased in thickness; and matter may be formed beneath the pericranium, in the same manner as in the corresponding affection of the periosteum elsewhere. In the second period, the disease has spread to the dura mater, which becomes thickened like the pericranium, and the inner surface of the bones is also rough and ulcerated; and the nerves, in their passage through their appropriate foramina, are pressed upon, by thickening of their own external coat, and by the irregular growth of the bones and their membranes.

The most striking instance which I have seen of this affection of the nerves, was one which I had an opportunity of witnessing in the Middlesex Hospital some years since, which has also been minutely described by my friend Mr. Mayo, in his "Physiological Commentaries." In this case the affection was confined to the left side of the face, which was œdematous to a considerable extent. The olfactory nerve on this side had lost its powers, and the optic nerve of the same side seemed to be impaired, as the sight of the eye became dim during the progress of the disorder, and the pupil did not change when a strong light was presented to the eye. The third, fourth, and sixth nerves were distinctly paralysed, as the eye was immovable, and the eyelid could not be raised. The muscular branches of the fifth nerve were also paralysed; for, if the person placed a piece of crust between his teeth, the masseter of the right side became hard, but that on the left remained flaccid. The branches of the same nerve which supplied the skin of the face had also lost their functions, since the only part of the face which retained its sensibility was a narrow line supplied by the second cervical nerve. The mucous surfaces of the eye, of the left nostril, and of the gums on the left side, had lost the sense of touch; and the tongue on the left side, when its surface only was irritated, was insensible to touch as well as to taste. The hearing was also deficient; so that the only nerves which at that time remained unaffected were the eighth and ninth pair. It was a curious circumstance that in this case the eye was much inflamed, and superficially ulcerated, possibly from the loss of power in the fifth nerve, as happened in the experiments of Magendie. These symptoms did not come on at once, but one nerve after another became affected, during the course of many months.

The ulceration of the bones in such cases is generally unattended with suppuration, but sometimes matter is secreted between the dura mater and the bone, in the same manner as it is formed in the soft nodes situated externally. In this case, unless an opening has been made by ulceration through the whole skull, so as to allow a passage for the matter to be discharged externally, the further progress of the disorder is necessarily cut short by symptoms of pressure upon the brain. But fortunately this internal node, as it might be called, is a rare occurrence in this disease: though we shall afterwards see that matter is often formed in this situation, when the bone is primarily diseased.

In the third stage of the disorder, if it has been allowed to proceed so far, characterised by convulsions and by diminished mental powers, we evidently perceive the extension of the disease to the brain itself, which is not in an active state of inflammation, but is only irritated by the disease of the dura mater and the bone; so that if the brain is examined in persons who have died of this disease, the only morbid appearances are the effusion of serum beneath the arachnoid membrane and in the ventricles, with slightly increased vascularity of the substance of the brain.

*Remarks.*—This species of pains is then of a most formidable character, and, unlike the former species, appears to be produced immediately by the absorption of a morbid poison, and may therefore with more propriety be styled syphilitic: but is there any peculiarity in the syphilitic affection of the periosteum, to justify us in resorting to the use of mercury in every case? At a time when every disease which was capable of being cured by mercury was, for that reason, thought to be of venereal origin,—and when the reverse of this test was thought to be equally well founded, (a mode of argument which has given rise to the absurd name Pseudo-syphilis,)—it was not surprising that every affection of the periosteum, such as I have attempted to describe, was considered to be venereal.

But there can now be no doubt that many local affections are produced by blows upon the periosteum, which nothing but the history of the complaint will enable us to distinguish from venereal nodes. In one case, after such a swelling had existed for three years in an indolent state, the occurrence of secondary symptoms of lues occasioned a fresh activity in the tumor, which was cured by the same means as the other symptoms. In Mr. Brodie's collection is the skull of a man, who had received a blow upon the head, which produced such an appearance in both surfaces of the bones, as I have described to arise from disease of the pericranium and the dura mater; so that no difference can be detected when this skull is placed by the side of another which is known to be syphilitic.

These cases also occur spontaneously. For instance, a woman was admitted into St. George's Hospital, under the care of Mr. Brodie, having for four years suffered from pain in the head, followed by fits and by diminution of the mental powers. The pain was general, but in one part the surface of the occipital bone was exposed, carious, but not dead. She was almost completely deaf, and had paralysis of the portio dura on one side, with a curious corrugation of the tongue, apparently arising from loss of power in the muscles of one side. A portion of bone was removed by the trephine, to which the dura mater partly adhered, but a small spiculum of bone was found on its surface, with a drop or two of pus. Several other cases have also fallen under my notice.

The fact that other morbid poisons, besides that of lues, produce general pains in the periosteum, is also well known, and by none are they caused more frequently than by mercury, when injudiciously employed. Mr. Travers, in his Essay on Iritis, alludes to this action of mercury as a circumstance well known to the profession; and I am also satisfied of the fact from my own observation, as well as from the assertions of others.

The formation of nodes in syphilis is a subject on which great difference of opinion exists. It is asserted, on the one hand, that nodes of the periosteum, constitutionally produced, (for of their local origin from blows there can be no doubt,) do not arise from the influence of mercury alone; while other practitioners, of equal experience, express their belief that, even after syphilis, they do not occur, unless mercury has been exhibited, and attribute their formation to this medicine alone, or to the joint action of mercury and syphilis. For my own part, I have no doubt, from many cases which have fallen under my notice, and a consideration of the alteration of structure which the periosteum and the bone undergo, that disease of the periosteum may arise from many different causes besides local injuries; and, as the progress of the complaint appears to be the same in all instances, that any morbid poison capable of producing the first stage of the disease in the periosteum, may terminate in the formation of local tumors or nodes.

It is fortunate, however, as Mr. Travers has observed with regard to the disputed origin of iritis, that, in this disease also, our being unable clearly to trace the disease to its origin is not of essential importance in the regulation of our treatment. Whether the disease arises, in any particular instance, from the action of the syphilitic poison, or from mercury, or from the effects of both conjoined, or from whatever cause it derives its origin, still the course of the disease is so similar, that the same general plan is to be adopted. There can be no doubt that idiopathic affections of the periosteum may sometimes be cured by mercury, at other times without this medicine: such is also the case in syphilitic instances of the disorder; and, like iritis, nodes clearly traced to the action of mercury may often be cured by a more extended or more judicious use of the same medicine.

*Treatment.*—In the treatment of this disorder, the only constitutional means which can be safely relied on is a course either of mercury or of sarsaparilla, or of both medicines combined; and, in deciding our choice, several circumstances —such as the constitution of the patient, the history of the complaint, the nature of other venereal symptoms which accompany the pains, and the appearance of the tumors,—are to be taken into consideration.

The peculiarities of constitution which prohibit the use of mercury altogether, or indicate the necessity of extreme caution in its use, are well known: such as the existence of a strumous or phthisical diathesis; a highly nervous temperament, disposing to the production of erethismus; a peculiar idiosyncracy with regard to mercury; or that state of the system which is often called Cachexia syphiloidea.

Where no other symptoms are present, mercury is generally unnecessary, except when some large nodes, without suppuration, are present; and we may often be induced to give mercury on account of other syphilitic symptoms, in which case the pains will get well at the same time with these symptoms, though they might, if alone, have been cured by other means.

As a general rule, it may perhaps be said, in the first place, that the employment of mercury is *advantageous*, as the quickest method of curing the existing complaints, and the most effectual means of preventing a relapse, when nodes are formed early in the complaint, (early with reference either to the first reception of the virus, or to the duration of the pains in the periosteum;) and when the nodes are of large size, and composed evidently of periosteum or bone without any suppuration, and without much inflammation, and are situated on the cylindrical bones.

Secondly, that mercury is *unnecessary* when the pains continue for some time without giving rise to any local swelling; or when the nodes still retain a semi-cartilaginous appearance, and give a similar sensation to the finger to that which may be observed when they appear only at intervals.

And, in the third place, that the use of mercury is by all means *to be avoided,* whenever the disease has extended to the bones in such a manner as to produce soft nodes containing matter; in which case this medicine will seldom be borne with impunity by the constitution, and the disease will often be aggravated by it. Sometimes, indeed, the mercurial action will at first be attended with very beneficial results; but the system appears unable to support its influence for a length of time sufficient to produce a complete cure, and the case suddenly recedes into a state fully as bad, or even worse, than before the mercury was begun. Whenever we are induced by peculiar circumstances to employ mercury in such cases, it ought therefore to be combined with sarsaparilla, or other tonics. The use of mercury is also attended with more risk when the pericranium is the seat of the disease, than where the periosteum of other bones is affected.

On the whole, I am inclined to believe that, in by far the majority of cases of this disease of the periosteum, mercury is unnecessary; and that there is scarcely any symptom of the venereal disease, in the treatment of which we are more indebted to the researches of Mr. Rose and other surgeons, than the one under consideration. But, although I would confidently rely upon the efficacy of sarsaparilla in almost every case, still it must be confessed that in some instances the cure will not be permanent: even in these, however, this medicine is a valuable auxiliary to mercury.

Of local remedies little need be said, as their utility is evident in cases of nodes, for which leeches, blisters, mercurial plasters, and other applications, are of considerable service. A simple incision through a node of the periosteum is another method of treatment, which is well known to relieve almost instantaneously the most acute pain. It seems to be effectual principally by taking away the tension from the membrane, but probably assists the subsequent absorption of the tumor by the suppuration which is thus established. It is not advisable that any exfoliation of the bone should be produced, which is recommended by some authors. The same plan is also applicable to the severe pain arising from thickening of the pericranium without nodes, although in such cases, from the extensive nature of the disease, the pain is sometimes apt to return. More than once, however, I have known a patient beg that the operation might be repeated, in consequence of the complete relief from pain which had been given by a previous division. Where the dura mater and inner surface of the bones of the cranium are affected, some external appearances will occasionally point out the particular seat of the disease, so as to justify the removal of a portion of bone by the trephine; an operation which I have seen practised several times, with material alleviation of the symptoms.

The propriety of opening nodes in which matter has been formed, is a point which admits of discussion, but the chances of absorption are so considerable that it should not be done hastily: sometimes, however, a small puncture made with a lancet at a proper time will prevent sloughing of the integuments, and the bone, even when ulcerated so as to feel quite dead on the introduction of the probe, will recover itself after the evacuation of the matter.

4. *Of the disease of the bones.*—In the preceding description of the disease of the periosteum, it has been found to extend secondarily to the external surface of the bones; syphilitic ulcers from eruptions may also slough so deeply as to destroy a portion of the bones situated below. This often happens in the tibia; and ulcers in the throat and pharynx will often produce exfoliation of the soft bones at the base of the skull, and those of the nose and palate. In one case of this kind, which occurred at the Lock Hospital, an ulcer in the pharynx produced disease of the ligaments and of the bony structure of the upper vertebræ; and the person died suddenly from the dentata having, from this cause, been allowed to fall in so as to crush the spinal marrow.

But a similar loss of vitality in a bone may arise from an ulcer produced by mercury, or by any other cause; so that in such cases the bone cannot strictly be said to be affected by lues.

Again, the cancellous structure of the extremities of the long bones, or those of the wrist and fingers and the sternum, frequently become affected during the progress of syphilis: they inflame, suppurate, and exfoliate, sometimes in many different parts of the body at the same time, and often while the skin is covered by syphilitic eruptions. But these swellings, also, it would be wrong to call syphilitic, since they occur only in scrofulous persons, and run exactly the same course as when the bones are similarly affected without any venereal taint. It is only an instance of the excitement of a dormant disease, by the irritation of the syphilitic virus upon a strumous constitution; in the same manner as the absorbent glands, or tubercles in the lungs, often become actively diseased from the influence of the same poison, or from mercury or any other debilitating cause.

The bones of the nose become, perhaps, most frequently diseased in the manner just alluded to; but sometimes, probably, from the direct action of syphilis upon them, and from ozœna or ulceration beginning primarily in the Schneiderian membrane. But my present limits will not allow of a full discussion of this subject, and the progress of caries and necrosis of these bones after syphilis is not materially different from similar affections arising from other causes. From whatever cause, however, they have become diseased, the employment of mercury will almost invariably be found prejudicial.

But there is another disease of the bones, which seems to possess some peculiar characters, and which may perhaps with more propriety be denominated syphilitic, although in most cases the cause of the disorder is so complicated that it is difficult to ascertain how much is to be attributed to the specific action of lues upon the bones, and how much to the joint influence of this disease and of mercury. A great part of the violence of syphilis, as it formerly prevailed, particularly the extensive honeycomb caries of the cranium, and the loss of the bones of the nose, (which may be seen in anatomical museums,) was, without doubt, owing to the empirical and destructive employment of mercury; but yet it seems to me to be probable that the disease I have now to mention originates, in many instances at least, from the direct influence of syphilis upon the bones.

The parts most subject to it are the tibia and the bones of the cranium. There is, in these cases, for a long time a very acute pain in some particular part of the bone, not diffused like the pain in the early stages of disease of the periosteum, but fixed to some defined spot, which the patient says he can cover with his finger, or that it is not larger than a half-crown, or some similar expression. Like the other kinds of pain, it is variable, and increased by the warmth of the bed. After a time, a soft tumor arises over the painful spot, containing an ill-conditioned kind of pus; the skin becomes very thin and purple, and ulcerates, exposing carious bone at the bottom of the ulcer. So far there appears little difference between this kind of node and the soft node arising from disease of the periosteum, except the more fixed situation of the pain, and the absence of the hard ring of periosteum which generally surrounds an opening into a node of this membrane. But a more minute examination will generally show their different origin. If the bone at the bottom of a node of the periosteum is examined with the probe, it is hard, and feels like a bone accidentally deprived of its covering; and, if uneven on the surface from ulceration, the appearance of the part still seems to show what might be called an irregular healthy action, and exfoliation is confined to the outer layer of the bone, except in the cranium, where the inner surface of the bones is under the same circumstances as the outer layer only of other bones. But in this kind of abscess the bone is evidently itself diseased: it is generally softened and more vascular than usual, and a central hole may be detected, through which the probe may be passed into the cancellous structure, or between the tables of the skull. From this opening unhealthy granulations often arise, which are exquisitely tender. Sometimes a fungus grows in this manner, before an external opening is formed, and, being confined by the periosteum, the most excruciating pain is produced whenever pressure is made, or when warmth causes a temporary increase in the size of the fungus. The portion of bone which is thus affected very frequently dies and exfoliates, leaving a deep hollow, which is not again filled up by new bone. Sometimes, when the syphilitic action is arrested, the separation is so slowly effected that the old skin, or newly-formed cicatrix, becomes adherent to the surface of the dead portion of bone, leaving the central opening uncovered, so that the probe may still be passed into the black sequestrum which is thus exposed.

The nature of this affection when it attacks the cranium is essentially the same as elsewhere: it originates in the diploe, and small holes are formed by ulceration through the tables; sometimes several of them are formed near each other, and thus produce that worm-eaten appearance which is so remarkable in these cases. Frequently ulceration takes place through both tables of the skull, particularly around a dead portion of bone, and pus is seen oozing out of the holes upon each pulsation of the brain, and again receding till the next impulse is given by the arteries. The dura mater is not, in general, so extensively thickened as in the disease of the periosteum, so that, when the pus has a free exit through the skull during the process of separation, the granulations upon the dura mater are healthy, and the patients frequently recover. In one case several deep holes remained after the separation of pieces of the occipital and parietal bones, over which perfect cicatrices had been formed; and, when I first saw the patient, the skin was closing in over another opening of the same kind. Besides these openings, nearly the whole of the frontal bone formed a large exfoliation, through which the dura mater was exposed in two places, and separation appeared, from the movement of the pus just alluded to, to be going on around the whole extent. This person suffered very little inconvenience from the disease, notwithstanding an accidental fall when in this situation, which caused slight symptoms of concussion, and produced considerable hemorrhage from the dura mater.

The most frightful example of the disease which I have witnessed, occurred in a man who died during the time I resided as house-surgeon at the Lock Hospital. His skull has been presented to the College of Surgeons; and a preparation has also been made of the scalp, to show the great extent to which it had been destroyed by ulceration. The disease of the bones reached into the orbits, so as to produce complete and disgusting eversion of the eyelids, terminating in total blindness. In this man, as well as in the former case, the brain was little disturbed by the great extent of the disease, till the last two months of his life, when frequent convulsions took place, with gradual loss of the mental faculties.

A cessation of pain for a considerable time before the appearance of matter, is not unfrequent in these cases; so that the death of a portion of bone is for a long time unsuspected, till the formation of abscess over it again directs the attention of the patient to the disease.

The disease of the bones which I have thus attempted to describe, is more formidable than either of the preceding diseases; it indicates a very depraved state of constitution, and is generally accompanied with extensive sloughing ulcers of the pharynx, and with sloughing or phagedenic ulcers on the body, from eruptions of rupia or ulcerating tubercles. Mercury scarcely ever agrees with persons labouring under this disease of the bones, and yet, from the obstinacy or peculiar nature of the concomitant symptoms, we are often obliged to employ it: its effects must, however, be very carefully watched, and the quantity very gradually increased; and sarsaparilla and other tonics should always be exhibited at the same time. To these latter medicines, which may often be combined with advantage with large doses of ammonia, the cure should generally be trusted in the first instance. An increase or return of pain in the bone during a mercurial course is a very bad sign, and points out the necessity of immediately desisting from its use.

The appropriate local applications, and the surgical treatment of exfoliations, will easily suggest themselves during the progress of each case.

31, *Half Moon-street; January* 1827.

